# DO PERSONALITY TRAITS INFLUENCE PERCEPTIONS OF COGNITIVE CHANGE IN COMMUNITY DWELLING OLDER ADULTS?

**DOI:** 10.1093/geroni/igz038.3267

**Published:** 2019-11-08

**Authors:** Thomas M Meuser, Regula H Robnett

**Affiliations:** University of New England, Portland, Maine, United States

## Abstract

Recent research has linked personality traits and risk for cognitive impairment in advancing age. Associations with neuroticism are particularly robust. Both longstanding and recent elevations may predict dementia. Other traits – conscientiousness and openness to experience – also show unique associations. These findings derive mainly from large sample population studies and smaller clinical investigations. Relevance to the general population is unclear. We investigated the “big five” personality traits and cognition in 232 community-dwelling adults (73% female, 97% Caucasian, mean age 72 years). Scores on a self-report screen for dementia – the AD8 – framed the sample: 77% scored 0 points, no dementia; 23% scored 2+, possible dementia. Age and personality were independent variables in a binary logistic regression with AD8 status as dependent. All predictors but one, extraversion, were significant (p < .05), suggesting that personality traits may influence perceptions of cognitive change. Higher agreeableness and neuroticism predicted possible dementia status on the AD8, whereas higher openness and conscientiousness predicted normal cognition. Interestingly, most in the AD8 positive group (70%) denied having “more problems with memory than most” on the Geriatric Depression Scale. These perceptions would seem incompatible, especially for true positive cases. Our findings suggest that the role of personality in dementia screening (and, perhaps, diagnosis) may be more nuanced than indicated in other studies. Longstanding traits and present perceptions are both elements of the evaluative process, as much as test scores and reported history. Our findings speak to the value of a person-centered, context-aware approach in cognitive screening.

